# Estimating the Cost of 3 Risk Prediction Strategies for Potential Use in the United Kingdom National Breast Screening Program

**DOI:** 10.1177/23814683231171363

**Published:** 2023-05-04

**Authors:** Stuart J. Wright, Martin Eden, Helen Ruane, Helen Byers, D. Gareth Evans, Michelle Harvie, Sacha J. Howell, Anthony Howell, David French, Katherine Payne

**Affiliations:** Manchester Centre for Health Economics, Division of Population Health, Health Services Research and Primary Care, School of Health Sciences, The University of Manchester, Manchester, UK; Manchester Centre for Health Economics, Division of Population Health, Health Services Research and Primary Care, School of Health Sciences, The University of Manchester, Manchester, UK; The Prevent Breast Cancer Research Unit, The Nightingale Centre, Manchester University NHS Foundation Trust, Manchester, UK; Division of Evolution and Genomic Science, The University of Manchester, Manchester, UK; Manchester Centre of Genomic Medicine, Manchester University NHS Foundation Trust, Manchester, UK; The Prevent Breast Cancer Research Unit, The Nightingale Centre, Manchester University NHS Foundation Trust, Manchester, UK; Manchester Centre of Genomic Medicine, Manchester University NHS Foundation Trust, Manchester, UK; Division of Evolution and Genomic Science, The University of Manchester, Manchester, UK; Manchester Academic Health Science Centre, Health Innovation Manchester, Manchester, UK; Division of Cancer Sciences, School of Medical Sciences, Faculty of Biology, Medicine and Health, The University of Manchester, Manchester, UK; Manchester Breast Centre, Manchester Cancer Research Centre, University of Manchester, Manchester, UK; NIHR Manchester Biomedical Research Centre, The University of Manchester and Manchester University NHS foundation trust; The Prevent Breast Cancer Research Unit, The Nightingale Centre, Manchester University NHS Foundation Trust, Manchester, UK; Manchester Academic Health Science Centre, Health Innovation Manchester, Manchester, UK; Division of Cancer Sciences, School of Medical Sciences, Faculty of Biology, Medicine and Health, The University of Manchester, Manchester, UK; Manchester Breast Centre, Manchester Cancer Research Centre, University of Manchester, Manchester, UK; NIHR Manchester Biomedical Research Centre, The University of Manchester and Manchester University NHS foundation trust; The Prevent Breast Cancer Research Unit, The Nightingale Centre, Manchester University NHS Foundation Trust, Manchester, UK; Division of Cancer Sciences, School of Medical Sciences, Faculty of Biology, Medicine and Health, The University of Manchester, Manchester, UK; Manchester Breast Centre, Manchester Cancer Research Centre, University of Manchester, Manchester, UK; NIHR Manchester Biomedical Research Centre, The University of Manchester and Manchester University NHS foundation trust; The Christie NHS Foundation Trust, Manchester, UK; The Prevent Breast Cancer Research Unit, The Nightingale Centre, Manchester University NHS Foundation Trust, Manchester, UK; Division of Cancer Sciences, School of Medical Sciences, Faculty of Biology, Medicine and Health, The University of Manchester, Manchester, UK; Manchester Breast Centre, Manchester Cancer Research Centre, University of Manchester, Manchester, UK; NIHR Manchester Biomedical Research Centre, The University of Manchester and Manchester University NHS foundation trust; The Christie NHS Foundation Trust, Manchester, UK; NIHR Manchester Biomedical Research Centre, The University of Manchester and Manchester University NHS foundation trust; Manchester Centre for Health Psychology, Division of Psychology and Mental Health, School of Health Sciences, Faculty of Biology, Medicine and Health, University of Manchester, Manchester, UK; Manchester Centre for Health Economics, Division of Population Health, Health Services Research and Primary Care, School of Health Sciences, The University of Manchester, Manchester, UK

**Keywords:** micro-costing, breast cancer screening, risk prediction, risk-based cancer screening

## Abstract

**Background:**

Economic evaluations have suggested that risk-stratified breast cancer screening may be cost-effective but have used assumptions to estimate the cost of risk prediction. The aim of this study was to identify and quantify the resource use and associated costs required to introduce a breast cancer risk-stratification approach into the English national breast screening program.

**Methods:**

A micro-costing study, conducted alongside a cohort-based prospective trial (BC-PREDICT), identified the resource use and cost per individual (£; 2021 price year) of providing a risk-stratification strategy at a woman’s first mammography. Costs were calculated for 3 risk-stratification approaches: Tyrer-Cuzick survey, Tyrer-Cuzick with Volpara breast-density measurement, and Tyrer-Cuzick with Volpara breast-density measurement and testing for 142 single nucleotide polymorphisms (SNP). Costs were determined for the intervention as implemented in the trial and in the health service.

**Results:**

The cost of providing the risk-stratification strategy was calculated to be £16.45 for the Tyrer-Cuzick survey approach, £21.82 for the Tyrer-Cuzick with Volpara breast-density measurement, and £102.22 for the Tyrer-Cuzick with Volpara breast-density measurement and SNP testing.

**Limitations:**

This study did not use formal expert elicitation methods to synthesize estimates.

**Conclusion:**

The costs of risk prediction using a survey and breast density measurement were low, but adding SNP testing substantially increases costs. Implementation issues present in the trial may also significantly increase the cost of risk prediction.

**Implications:**

This is the first study to robustly estimate the cost of risk-stratification for breast cancer screening. The cost of risk prediction using questionnaires and automated breast density measurement was low, but full economic evaluations including accurate costs are required to provide evidence of the cost-effectiveness of risk-stratified breast cancer screening.

**Highlights:**

- Economic evaluations have suggested that risk-stratified breast cancer screening may be a cost-effective use of resources in the United Kingdom.
- Current estimates of the cost of risk stratification are based on pragmatic assumptions.
- This study provides estimates of the cost of risk stratification using 3 strategies and when these strategies are implemented perfectly and imperfectly in the health system.
- The cost of risk stratification is relatively low unless single nucleotide polymorphisms are included in the strategy.

There is growing interest in the use of strategies to predict a woman’s future risk of developing breast cancer using combinations of factors as an approach that may help to improve the balance of risks and benefits in national breast screening programs for the early detection of breast cancer.^1^ A multitude of factors are known to contribute to a woman’s risk of developing breast cancer, including a family history of breast cancer, age, weight gain from early adulthood, age of menarche, whether she has been prescribed hormone replacement therapy, and age at which she had children (if any). The density of a woman’s breast tissue also affects her risk of breast cancer, with those with denser breasts at higher risk.^2^ Genetic factors also play a role, with pathological variants in several moderate- to high-risk genes including *BRCA1*, *BRCA2*, and *PALB2* substantially increasing a woman’s risk of breast cancer. In addition, genome-wide association studies have identified hundreds of single nucleotide polymorphisms (SNPs) that may influence a woman’s risk.^3,4^

A number of risk prediction models have been developed based on clinical research, examples of which include the Tyrer-Cuzick, BOADICEA, and Gail models.^5–7^ The rationale underpinning the use of a risk-based approach is to identify more cancers at an earlier stage for those at high risk while mitigating the potential harms of breast cancer screening, by accounting for individual risk profiles in the population of eligible women. For example, those at higher risk of breast cancer might benefit from more frequent screening to aid early identification. Those women predicted to be at lower risk might benefit from a lower frequency of breast cancer screening. Targeted breast cancer screening may also have economic benefits. Resources used in screening programs could be optimally allocated according to individual risk profiles diverting health care resources from women at lower risk to more intense screening for women at a higher than population risk of breast cancer.

A number of research studies, including the Women Informed to Screen Depending On Measures of risk (WISDOM) and My Personalised Breast Screening (MyPebs) studies, have been established to evaluate risk-stratified screening programs.^8,9^ The Predicting Risk of Cancer at Screening (PROCAS) study conducted within the UK National Health Service National Breast Screening Programme (NHSBSP) provided evidence that risk-stratified screening is likely to identify more cancers in high-risk women and importantly is acceptable to patients.^10^ Using the results of the PROCAS study, an automated system for estimating women’s risk of cancer at their first screen, known as BC-PREDICT, was developed. Real-time risk feedback (within 6–8 wk) was provided following self-completion of the Tyrer-Cuzick (TC) questionnaire, mammographic density assessed through Volpara TruDensity, and in a proportion of women saliva DNA testing using a panel of 142 SNPs.

The risk-prediction strategy used in BC-PREDICT produced estimates of a woman’s 10-y and lifetime risk of developing breast cancer. Those women considered at high risk of developing cancer in the next 10 y (≥8% predicted risk) were offered annual mammography screening and a discussion about preventative medicine to reduce their cancer risk. Women between the ages of 47 and 49 y at moderate risk of breast cancer in the next 10 y (3%–7.9% predicted risk) were offered annual screening for those years and then 3 yearly screenings over age 50 and the potential for the use of preventative medicines discussed.

The harms and benefits of BC-PREDICT and the resulting changes in women’s recommended screening interval are currently being evaluated compared with the current UK practice of standard 3 yearly screening.^11^ Approaches to risk stratification need to be both effective in improving outcomes and a cost-effective use of the health care budget before they can be recommended for implementation. Economic evaluation is used to determine the cost-effectiveness of new health care initiatives by synthesizing associated health costs and consequences. A previous economic modeling evaluation of a risk-stratified screening strategy indicated that it is a potentially cost-effective use of resources.^12^ However, in this study, the cost of the risk-stratification strategy was estimated using a range of pragmatic assumptions. As the incremental cost-effectiveness of the stratified screening programs evaluated depended on the cost of stratification, producing a more accurate estimate of the cost of stratification is important to reduce uncertainty about whether such screening programs represent an efficient use of health system resources.

The aim of this study was to identify and quantify the resource use and associated costs required to introduce a breast cancer risk-stratification approach into the English national breast screening program.

## Methods

A micro-costing study assumed the NHS (service provider) perspective to identify the resource use and cost per individual (£; 2021 price year) of providing a risk-stratification strategy at a woman’s first mammography as part of a national population-level screening program (hereafter “risk-stratification for NHSBSP”). After consultation with the University of Manchester Research Ethics office, it was deemed that this study did not require ethical approval, as it was both a service evaluation and research involving interviews with participants on subjects deemed to be within their professional competence.

Micro-costing involves the detailed recording (bottom-up) of all resource use to ensure transparency and accuracy. Following the approach by Jani and colleagues,^13^ this micro-costing study involved the following defined stages (see Supplementary Appendix 1): identifying the risk-stratification pathway, identifying the resources used in the risk-stratification pathway, identifying unit costs for the resource use, and data analysis.

### Identifying the Risk-Stratification Pathway

The risk-stratification pathway was identified following discussions with a consultant oncologist and the project principal investigator for the BC PREDICT study. An iterative interview-based process based on input from a clinical trials assistant (CTA) and geneticist also involved in the study was used to refine the pathway.

### Identifying the Resources Used in the Risk-Stratification Pathway

To identify resource use in the risk-stratification strategy, semistructured interviews were conducted with 2 CTAs, a medical oncologist, and a geneticist involved in the study. Interview schedules (see Supplementary Appendix 4) were created using the risk-stratification pathway as a guide, and these experts were asked to reflect on whether there were any costs that had not been discussed or any other implementation issues that they had personally experienced.

Five interviews were conducted with individuals who had been involved in conducting the BC-PREDICT study to estimate the proportion of participants who followed specific routes within the pathway. These experts comprised a medical oncology consultant, a consultant geneticist, 2 CTAs, and a research assistant in genetics.

### Identifying Unit Costs

Unit costs were derived from a number of sources. Staff costs were taken from the Personal Social Services Research Unit (PSSRU) Unit Costs of Health and Social Care 2021 report.^14^ The costs associated with University of Manchester eMarketplace of approved suppliers. The cost of the OncoArray SNP kits were taken from quotes generated by Illumina. Other sources of costs included NHS Healthcare Resource Group data. Where no published unit costs were available, best estimate assumptions were used (see Tables 1 and 2). Where required, costs were inflated to 2021 values using the NHS Hospital and Community Services index (up to 2016) and NHS cost inflation index (2016–2021), which represent health service specific inflation.

**Table 1:** Resources Required to Provide the Risk-Stratification Strategy in the NHS

Resource Type	Resource Use	Sources and Assumptions	Unit Cost	Total Cost per Woman (£)	Unit Cost Sources and Assumptions (Reference)
Sending invitation to women to have their breast cancer risk predicted					
Printing cost of study invitation and participant information	7 sheets of paper	Interviews with 2 CTAs	12p per sheet	0.84	(Ulph et al.^15^) Inflated to 2021 levels
Staff time printing letter and filling envelope	5 min	Interviews with 2 CTAs Mean of midpoints provided. One admin stated 2 min, and the other stated 42 in a half a day to a day. In a 7.5-h day, this implied 5.36 to 10.71 min with a midpoint of 8.03	£35 per hour	2.92	(Jones and Burns^14^) PSSRU 2021, hourly cost of grade 4 “scientific staff” as proxy for administrator (not included)
Postage cost of sending the invitation	1 second-class stamp	Assumption by research team	66p per survey	0.66	2021 cost of a UK second-class stamp
Tyrer-Cuzick questionnaire					
Printing cost of paper surveys	4 sheets of paper	Interviews with 2 CTAs	12p per sheet	0.48	(Ulph et al.^15^) Inflated to 2021 levels
Postage cost of returning paper surveys	1 second-class stamp	Assumption by research team	66p per survey	0.66	2021 cost of a UK second-class stamp
Administrative staff time to transfer data from paper survey to CRA computer system	21.25 min	Interviews with 2 CTAs Mean of midpoints of time ranges provided (12.5 [10–15 min] and 30 [0 to 60 min from “up to one hour”])	£35 per hour	12.40	(Jones and Burns^14^) PSSRU 2021, hourly cost of grade 4 “scientific staff” as proxy for administrator (not included)
Volpara breast density analysis					
Volpara analysis	1 per woman	—	£1.15 per measurement	1.15	Personal communication US $1 to $2 per measurement
Implementation issues with Volpara breast density measurement					
Staff time following up failed image transfers	6.25 min	Interviews with 2 CTAs Mean of midpoint from ranges provided (7.5 and 5 min)	£62 per hour	6.46	(Jones and Burns^14^) PSSRU 2021, hourly cost of grade 7 “scientific staff” as proxy for research fellow (not included)
Staff time providing manual BI-RADS assessment of failed mammography image transfers	1 min	Personal communication with administrator and statistician on project	£123 per hour	2.05	(Jones and Burns^14^) PSSRU 2021, medical consultant
Uninstalling and reinstalling internet aerial on movement of screening van	NHS breast screening regions conduct 580 mammograms per week. It is assumed on average that 2 screening vans operate in each trust and move on average every 3 wk.	Average number of screens per breast screening region from (NHS Digital^16^) Number of vans per trust and number of weeks before move based on researcher assumptions. These values are varied in the sensitivity analysis.	£500 per move	0.57	Personal communication
SNP saliva test					
Staff time to collect sample	3.5 min	Personal communication (midpoint of range of 2–5 min)	£41 per hour	0.68	(Jones and Burns^14^) Grade 5 nurse
Staff time to run SNP test	38.39 min (batch of 96) to 2,165 min (batch of 1) See Supplementary Appendix 5	Personal communication	£41 per hour	25.25 (for a full batch)	(Jones and Burns^14^) Grade 5 scientific staff
Consumables for SNP test	See Supplementary Appendix 5	Personal communication	Various See Supplementary Appendix 5	51.73 (for a full batch)	University of [redacted for anonymization] purchasing catalog
Generating and sending the risk letter					
Staff time retrieving data from CRA and transferring to collator	7.5 min	Interviews with 2 CTAs Midpoint of provided range (5–10 min)	£35 per hour	4.38	(Jones and Burns^14^) PSSRU 2021, hourly cost of grade 4 “scientific staff” as proxy for administrator (not included)
Printing cost of risk letter	2 sheets	Interviews with 2 CTAs	12p per sheet	0.24	(Ulph et al.^15^) Inflated to 2021 levels
Staff time printing letter and filling envelope	2 min	Interviews with 2 CTAs	£35 per hour	1.17	(Jones and Burns^14^) PSSRU 2021, hourly cost of grade 4 “scientific staff” as proxy for administrator (not included)
Postage cost of sending the risk letter	1 second-class stamp	Assumption by research team	66p per survey	0.66	2021 cost of a UK second-class stamp
Woman receives risk feedback and organizes risk consultation					
Staff time to organize risk appointment	2.25 min	Interviews with 2 CTAs Mean of midpoints of provided ranges (1.5 min and 3 min)	£35 per hour	1.31	(Jones and Burns^14^) PSSRU 2021, hourly cost of grade 4 “scientific staff” as proxy for administrator (not included)
Staff time to collate risk pack for health care professional	17.5 min	Interviews with 2 CTAs Mean of values provided (5 min and 30 min)	£35 per hour	10.21	(Jones and Burns^14^) PSSRU 2021, hourly cost of grade 4 “scientific staff” as proxy for administrator (not included)
Staff time for risk feedback appointments with women at high risk when implemented as in the study	30 min	Interview with consultant oncologist	£123 per hour	61.50	(Jones and Burns^14^) PSSRU 2021, medical consultant
Staff time for risk feedback appointments with women at moderate risk when implemented as in the study	20 min	Interview with consultant oncologist	£123 per hour	41.00	(Jones and Burns^14^) PSSRU 2021, medical consultant
Staff time for risk feedback appointments with women at high risk when implemented as in NHS	30 min	Interviews with 2 CTAs	£41 per hour	20.50	(Jones and Burns^14^) PSSRU 2021, Band 5 nurse
Staff time for risk feedback appointments with women at moderate risk when implemented as in NHS	20 min	Interviews with 2 CTAs	£41 per hour	13.67	(Jones and Burns^14^) PSSRU 2021, Band 5 nurse

BI-RADS, Breast Imaging Reporting and Data System; CRA, Cancer Risk Assessment; CTA, clinical trials assistant; NHS, National Health Service; PSSRU, Personal Social Services Research Unit; SNP, single nucleotide polymorphism.

**Table 2:** Probabilities of Patients Experiencing Different Events in the Pathway

Probability	Value	Sources and Assumptions
Probability of participants completing the TC questionnaire on paper v. online	0.0500	Interviews with 2 CTAs 10 paper surveys out of 200 total
Probability that a woman is predicted to be at high risk	TC: 0.0147 TC+VBD: 0.05 TC+VBD+SNP: 0.074	(Brentnall et al.,^3^ Evans et al.^17^)
Probability that a woman is predicted to be at moderate risk	TC: 0.0846 TC+VBD: 0.112 TC+VBD+SNP: 0.105	(Brentnall et al.,^3^ Evans et al.^17^)
Probability that a woman at high risk organizes a risk appointment	0.7429	(Evans et al.^17^)
Probability a woman at moderate risk organizes a risk appointment	0.7312	(Evans et al.^17^)
Probability a paper survey includes unclear data requiring telephone follow-up	0.3500	Interviews with 2 CTAs 3 to 4 out of 10
Probability a mammography image fails to send for breast density measurement	0.3250	Interviews with 2 CTAs Mean of estimates from 2 interviews (25% and 40%)
Probability a mammography image will require manual BI-RADs reading by a radiologist after Volpara image transfer failure	0.0450	Personal communication from administrator and statistician on project 85 mammography images out of 2,000 women

BI-RADS, Breast Imaging and Reporting Data System; CTA, clinical trials assistant; SNP, single nucleotide polymorphism; TC, Tyrer-Cuzick; VBD, Volpara breast density.

### Data Analysis

The expected costs of the risk-stratification strategy for women attending population screening were calculated based on observed resource use in the BC-PREDICT study. The expected costs of the intervention were reported for risk stratification using the Tyrer-Cuzick questionnaire alone (TC), the Tyrer-Cuzick and Volpara breast density measurement (TC+VBD), and the Tyrer-Cuzick, Volpara breast density, and SNPs (TC+VBD+SNP). In addition, the expected cost per woman of providing the risk stratification in an NHSBSP was calculated when the strategy was implemented in the NHS without the implementation barriers found in the study. The parts of the risk-stratification pathway contributing most to the cost of risk-stratification were identified and reported.

### Quantifying Uncertainty in Cost Estimates

To incorporate uncertainty in the estimates of the cost of the risk-stratification strategy, a deterministic sensitivity and probabilistic analysis was undertaken. The deterministic sensitivity analysis sought to explore the impact of differences in the resource use reported by the clinical trial assistants on the total cost of the different risk stratification strategies. The total costs of the risk stratification strategies were determined using the resource use reported by each CTA separately, and these were compared with the results when using the mean. Probabilistic analysis involves quantifying the joint uncertainty by assigning possible ranges and distributions to each parameter input used in the analysis. This analysis used the data and ranges provided in the semistructured interviews, and distributions were attached to the probabilities and resource use in the risk-stratification pathway (see Supplementary Appendix 5). Monte Carlo simulation was used to rerun the analysis multiple times, redrawing different parameter values from the distributions in each iteration. Checks of the results and their confidence intervals after different numbers of simulations suggested that 10,000 Monte Carlo simulations were sufficient to achieve stable results. The resulting sample of expected costs for each analysis group were used to generate pseudo confidence intervals around the point estimates of costs.

#### Scenario analysis to account for different laboratory batch sizes

To reflect the potential impact of partial batch sizes during a rollout of testing on the cost of risk stratification, a scenario analysis was undertaken to explore the impact of different SNP batch sizes on the cost of risk stratification for each strategy. In the main analysis, it was assumed that complete batches were used when conducting the genetic testing. This is a reasonable assumption because in the study, the researchers could wait until a batch was full to process the tests, and in clinical practice, there is likely to be enough testing demand to ensure batches are full or nearly full. A single OncoArray BeadChip kit costing £1,343.77 in the study could hold up to 48 tests. In addition, there are various other consumables used in the testing process that are bought in boxes of a set size, and some aspects of setting up the test such as DNA extraction are also conducted in batches of a certain size. These factors combine to mean the cost per SNP will be highly dependent on the number of tests in the batch.

## Results

**Figure 1:**
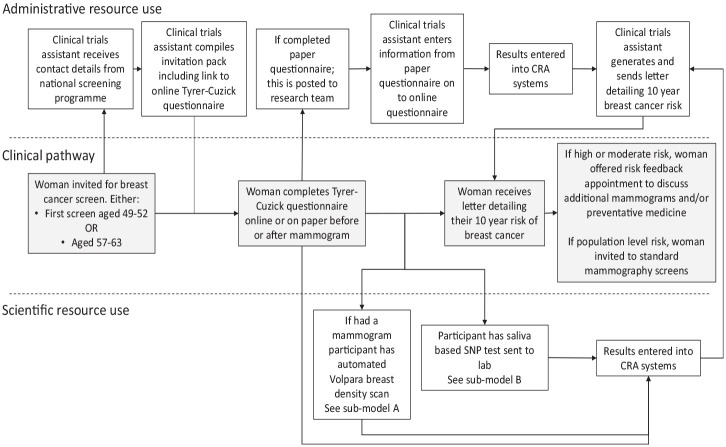
Pathway and resource use associated with risk-stratification in a breast cancer screening programme^1^.

The pathway diagram for the risk-stratification strategy implemented in an NHSBSP as explored in the BC-PREDICT study is shown in Figure 1. The diagram illustrates the pathway for patients and the administrative, imaging, and genetics support required in the risk-stratification strategy.

The pathway begins with a woman being invited to her first breast cancer screen as part of the NHSBSP, generally between the ages of 49 and 52 y.^i^ The team of CTAs received details of these women. From these details, they compiled an invitation pack including study details, consent forms, and a link to complete the TC risk questionnaire online. If an online TC was not completed, a paper version was offered at the screening appointment. Completed forms were subsequently posted to the study team and the information manually entered on the Cancer Risk Assessment (CRA) health system, a computer program that collates risk data from different sources into a central database. Online TC questionnaire data were automatically transferred to the CRA system with no additional administrative input. Mammography images were captured and sent to Volpara Health for automatic assessment with their Volpara Trudensity system, which uses x-ray physics and machine learning.^18^ The Volpara breast density (VBD) data were then automatically uploaded into the CRA system.

**Figure 2:**
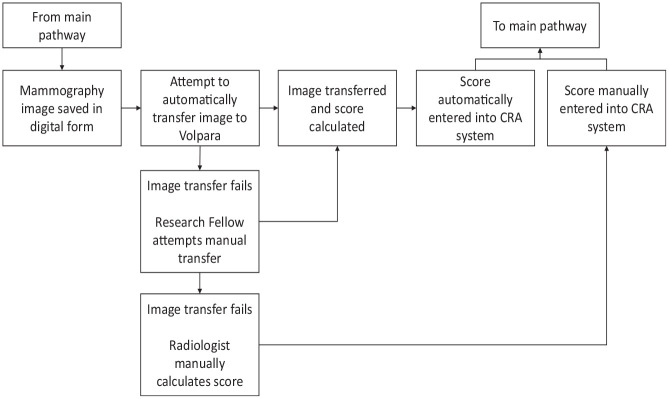
Pathway and resource use for obtaining breast density images (sub-model A).

In the BC-PREDICT study, there were a number of implementation issues with the use of the automated VBD measurement (see Figure 2; submodel A shown in Figure 1). Using Volpara required that mobile breast screening vans be fitted with aerials to enable internet access, whereas this was not previously required for standard mammography. A team of individuals including engineers was needed to fit the aerials, and each time the van was moved to a different site, the aerial had to be lowered and then raised at the new site. Even when the aerials were installed, there were issues with the automated transfer of mammography images to Volpara, and for a significant proportion of the time, these had to be manually sent by a research fellow working on the study. On some occasions, it was not possible to generate a breast density score, and the images had to be sent to a radiologist to manually read and complete a Breast Imaging Reporting and Data System (BI-RADS) estimation of breast density.

**Figure 3:**
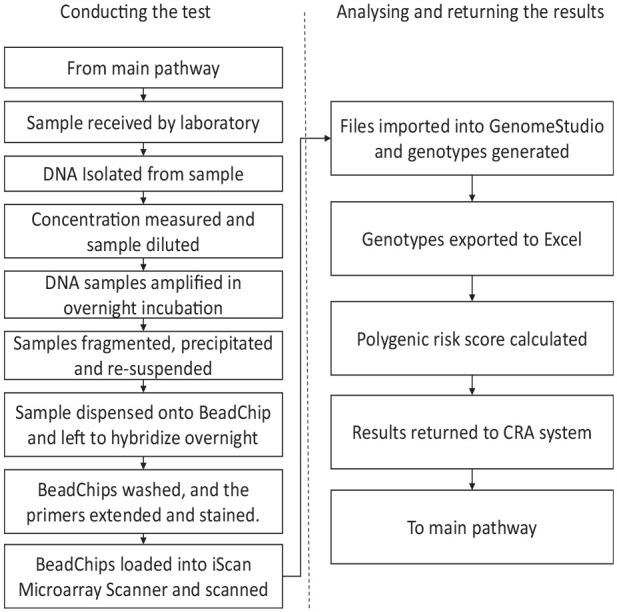
Pathway and resource use for obtaining genetic data (sub-model B).

In addition to the TC questionnaire and VBD measurement, a subsample of women in BC-PREDICT were offered a saliva DNA analysis to generate a polygenic risk score based on a panel of 142 SNPs. Full details about the included SNPs are available in Brentnall et al.^3^Figure 3 (submodel B from Figure 1) represents the process for preparing and analyzing samples. Further details of the testing process for SNPs are available in Supplementary Appendix 2. Upon completion of testing, the results were uploaded into the CRA system.

After the risk data had been entered into the CRA system, the CTA generated a letter detailing each woman’s risk and inviting individuals at high or moderate risk to an appointment at the Family History Risk and Prevention Clinic to discuss their risk status and potential actions that could be taken. If women wanted to take up the offer of a risk feedback consultation, they telephoned a CTA to arrange an appointment. Prior to a woman’s risk consultation, the CTA manually produced a pack detailing the woman’s risk of breast cancer and the contributing risk factors. The risk feedback appointments in the BC-PREDICT study were conducted with consultant medical oncologists ([redacted for anonymization]) or a consultant geneticist ([redacted for anonymization]). Potential actions approved by the National Institute for Health and Care Excellence (NICE) included inviting women to more frequent screening appointments and discussing the potential use of medicines for cancer prevention. Women at population-level risk were advised to attend screening at the normal interval of every 3 y via the NHSBSP.

### Moving from Research to National Rollout

While the pathway for the use of a risk-stratification strategy in an NHSBSP was informed by the experiences of the BC-PREDICT research team, it was anticipated that if the strategy was implemented in clinical practice as part of an NHSBSP, there would be modest adaptations to the way in which it was delivered, potentially affecting its cost. Supplementary Appendix 3 details the assumed changes in the delivery of risk prediction in the health service.

#### Resources used in the risk-stratification pathway

Table 1 summarizes the resources required to provide the risk-stratification strategy in the NHS setting. Table 2 shows the results from the interviews with experts that identified the flow of patients through the identified pathways based on the resources available.

#### The cost of risk stratification in a NSBP

The expected cost per woman receiving the risk-stratification strategy in each implementation setting (BC-PREDICT study or NHS practice) is shown in Table 3. A more detailed breakdown of the component elements of the cost of the stratification pathway is provided in Supplementary Appendix 7. The cost of providing the risk-stratification strategy at a woman’s first attendance at breast cancer screening in the BC-PREDICT study was calculated to be £16.45 for the Tyrer-Cuzick survey, £21.82 for the Tyrer-Cuzick with VBD measurement, £102.22 for the Tyrer-Cuzick with VBD measurement and genetic testing (SNPs). Key cost drivers in these strategies were generation and sending of the risk letter (43% of costs for Tyrer-Cuzick survey and 33% of Tyrer-Cuzick with VBD measurement), the manual sending of study invitations to women (27% of Tyrer-Cuzick survey and 20% of Tyrer-Cuzick with VBD measurement), and the organization and conducting of the risk consultation (25% of Tyrer-Cuzick survey and 32% of Tyrer-Cuzick with VBD measurement).

**Table 3:** Expected Cost of the Risk-Stratification Strategy in the Different Exemplar Applications and Implementation Settings

Strategy	Expected Cost^a^		
	Tyrer-Cuzick Survey	Tyrer-Cuzick Survey and Volpara Breast Density	Tyrer-Cuzick Survey, Volpara Breast Density, and Genetic Testing (SNP Analysis)
NHSBSP as in BC-PREDICT study	£16.45	£21.82	£102.22
NHSBSP in the NHS setting	£4.68	£6.64	£86.30

a £: 2021 prices.

The cost of adding in genetic testing using SNP analysis accounted for a large proportion of overall cost (78% in the BC-PREDICT study and 92% of Tyrer-Cuzick with VBD measurement and genetic testing [SNPs] in the NHS setting). When the implementation issues experienced in the study were assumed not to be relevant, the cost of risk stratification was calculated to be £4.68 for the Tyrer-Cuzick survey, £6.64 for the Tyrer-Cuzick with VBD measurement strategy, and £86.30 for the Tyrer-Cuzick with VBD measurement and genetic testing (SNPs) strategy. In the Tyrer-Cuzick survey and Tyrer-Cuzick with VBD measurement strategies used in the NHS setting for the breast cancer screening program, the key cost drivers were the generation and sending of the risk letter (58% and 41% of the cost, and the organizing and conducting of the risk feedback consultation [23% and 28% of cost]).

#### Quantifying uncertainty

The results of the deterministic sensitivity analysis in which the total costs of the risk stratification strategies were calculated for each CTA separately are reported in Appendix 8. While the resource use reported by each CTA varied widely, this did not result in large variations in total costs for the strategies. For example, the TC+VBD+SNP strategy in the trial had a mean expected cost of £102.22, with a total cost using the resource use of the first CTA of £99.33 and a cost of £105.13 when using the resource use reported by the second CTA.

Table 4 shows the mean expected cost, generated using probabilistic analyses, of the risk stratification when used in the different exemplars and when implemented as in the BC-PREDICT study or in the NHS setting.

**Table 4:** Results of the Probabilistic Analysis

Strategy	Estimated Cost^a^ (Pseudo–Confidence Interval)^b^		
	Tyrer-Cuzick Survey	Tyrer-Cuzick Survey and Volpara Breast Density	Tyrer-Cuzick Survey, Volpara Breast Density, and Genetic Testing (SNP Analysis)
NHSBSP as in BC-PREDICT	£15.26 (CI: £11.95 to £20.28)	£20.56 (CI: £16.51 to £26.47)	£101.10 (CI: £95.95 to £107.86)
NHSBSP in the NHS	£5.49 (CI: £4.90 to £6.47)	£8.46 (CI: £7.53 to £9.62)	£88.87 (CI: £85.92 to £92.29)

a £; 2021 prices.

b Pseudo–confidence intervals represent the cost of the intervention at the 2.5th centile and the 97.5th centile.

#### Scenario analysis

**Figure 4:**
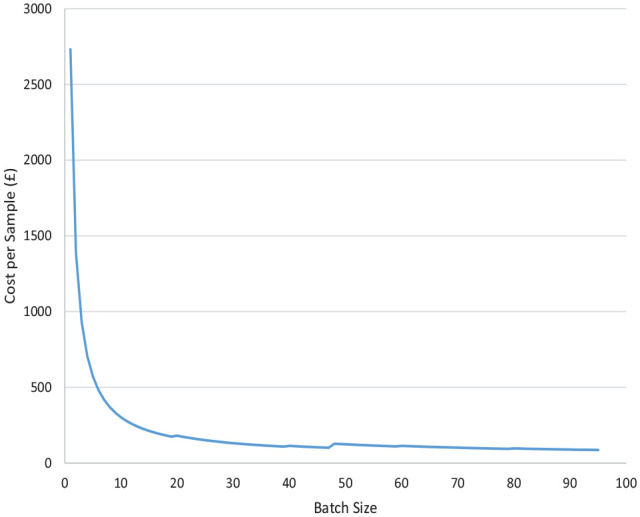
Cost of genetic testing (SNP analysis) by batch size.

Figure 4 shows the cost per analysis when genetic testing is conducted given the number of tests included in a batch. When an optimal batch size of 95 samples and 1 control sample is used, the cost of the TC+VBD+SNP strategy is £86.30, which rises to £2,731.98 when only 1 test is conducted. Increasing the batch size initially causes the cost per test to quickly fall. However, at a batch size of 48, the cost per test increases again, as a second OncoArray chip must be purchased to run the analysis. A higher cost per test is present until batch sizes exceed 71 tests. The per-sample cost remains less than £100 for batch sizes of 72 or more.

## Discussion

This micro-costing study calculated the cost of 3 exemplar strategies for risk stratification as part of a breast screening program. The use of the Tyrer-Cuzick survey alone or in combination with a VBD measurement was relatively inexpensive. The addition of genetic (SNP analysis) testing substantially raised the cost of all of the stratification strategies. Reducing batch sizes when conducting genetic testing led to increasing costs of testing up to £2,732 for a single test.

The cost of each of the exemplar strategies for risk stratification was substantially higher when implementation issues that had been faced in the BC-PREDICT study were taken into account. If the intervention was to be implemented in the health system, it would be important to address these issues to avoid unnecessarily using resources that could be better used to provide health benefits for other patients in the system. Putting in place implementation initiatives to better facilitate the transition of the risk-stratification strategy from research to clinical practice may be valuable.

The quantification of the difference in costs between a perfectly and imperfectly implemented strategy conducted in this study may help to establish a ceiling value on the benefits of strategies to improve the implementation of the intervention. For example, in 2019 to 2020, approximately 240,000 women received their first mammography screen as part of the English breast cancer screening program. If a strategy using the Tyrer-Cuzick survey and VBD were implemented, then the estimated cost of the strategy, assuming full uptake, would be £1.6 million per year if perfectly implemented but £5.2 million per year if the issues identified in the study remained present when the intervention was implemented in clinical practice. Failing to address these implementation issues may mean fewer funds are available for other interventions to prevent or treat cancer, and the difference in the total cost suggests that implementation initiatives to overcome these issues may be worth up to £3.6 million per year. This represents the value of perfect implementation.^19–21^ In reality, any implementation initiatives are unlikely to completely remove the barriers, and instead, the benefit would need to be estimated as the value of actual implementation.^19^

To determine whether risk-stratified screening using the risk-stratification strategy represents a good use of resources, an economic evaluation is required in which universal and targeted screening programs (i.e., comprising a risk-stratification strategy) are compared. A previous economic evaluation of risk-stratified breast cancer screening used assumptions from experts to estimate the cost of risk stratification using the Tyrer-Cuzick survey and VBD measurement as £10.57 (2017 GBP).^12^ At this cost, a strategy stratifying breast cancer screening by separating women into risk tertiles was predicted to be a cost-effective use of resources. In this study, the cost of the Tyrer-Cuzick survey and VBD measurement risk-stratification strategy was predicted to be slightly lower at £8.46 (CI: £7.53–£9.62), suggesting that risk-stratified screening using this approach is still likely to be cost-effective, assuming all of the other model parameters remain constant. Currently, no economic evaluation has sought to determine whether adding genetic testing using SNP analysis to a risk-stratification strategy in breast cancer is likely to be cost-effective. The additional cost of adding genetic testing would mean that the strategy might have to provide a substantial improvement in risk prediction and health benefit to be cost-effective. This may be possible if a reduction in screening is implemented in a low-risk group of <1.4% 10-y risk, as the proportion identified as low risk increases substantially from 4.7% to 24.9% by adding SNP313 to breast density and Tyrer-Cuzick data.^22^

There were some limitations to this study. Resource use was estimated from interviews with a relatively small sample of individuals. In addition, the resource use included in the study was based on that stated by the interview participants as opposed to observing resource use in practice. Stated resource use may suffer from issues of reliability, including participants’ ability to accurately recall how much time was spent undertaking given tasks and the tendency for participants to provide estimates in terms of certain time intervals, for example, 5 or 10 min. A strength of the study was that these individuals were experts in the topic, with extensive experience in using risk-prediction strategies as part of the BC-PREDICT study. When attaching unit costs, no costs were available for administrative staff in salary bands 2 or 3—those most likely to be involved in the provision of risk stratification. Instead, costs for band 4 scientific staff were used as a proxy, potentially resulting in a slight overestimation of the cost of risk stratification.

The implementation issues included in this costing study were those identified by the experts during the interviews. These issues arose in the research study evaluating the risk-stratification strategy, but it is possible that there may be a broader range of issues when attempting to implement the strategy in the health system. A more formal exploration of these potential issues using methods grounded in implementation science, for example, frameworks to identify barriers and facilitators, may provide a more comprehensive picture of the potential issues when introducing a risk-based NHSBSP.^23^

## Conclusion

Accurate and transparent costs for breast cancer screening risk-stratification strategies have been determined. Adding genetic testing to breast cancer risk stratification substantially increases costs. Implementation issues and their associated impact in increasing costs have been identified, providing useful information to inform the roll out of risk-prediction strategies as part of a national program in the NHS. Requisite evidence to facilitate subsequent economic evaluations of breast cancer screening programs has been provided.

## Supplemental Material

sj-docx-1-mpp-10.1177_23814683231171363 – Supplemental material for Estimating the Cost of 3 Risk Prediction Strategies for Potential Use in the United Kingdom National Breast Screening Program Click here for additional data file. Supplemental material, sj-docx-1-mpp-10.1177_23814683231171363 for Estimating the Cost of 3 Risk Prediction Strategies for Potential Use in the United Kingdom National Breast Screening Program by Stuart J. Wright, Martin Eden, Helen Ruane, Helen Byers, D. Gareth Evans, Michelle Harvie, Sacha J. Howell, Anthony Howell, David French and Katherine Payne in MDM Policy & Practice

## Notes

i In an expansion to the BC-PREDICT trial, women between the ages of 57 and 63 y attending screening were also offered the risk-stratification strategy.
