# Supplementary material for: Estimating the Cost of 3 Risk Prediction Strategies for Potential Use in the United Kingdom National Breast Screening Program
Source: MDM Policy Pract. 2023 May 4;8(1):23814683231171363. doi: 10.1177/23814683231171363 (PMC10161319; doi:10.1177/23814683231171363)
Supplement: sj-docx-1-mpp-10.1177_23814683231171363 – Supplemental material for Estimating the Cost of 3 Risk Prediction Strategies for Potential Use in the United Kingdom National Breast Screening Program [file sj-docx-1-mpp-10.1177_23814683231171363.docx]

Supplementary Appendices

**Estimating the cost of three risk prediction strategies for potential use in the United Kingdom National Breast Screening Programme**

Supplementary Appendix 1: Stages taken in costing the exemplar applications of breast cancer risk-stratification

Supplementary Appendix 2: Process of single nucleotide polymorphism (SNP) testing

Supplementary appendix 3: Assumed change in risk prediction in the health service compared to the trial

Supplementary appendix 4: Interview schedule

Supplementary appendix 5: Distributions of parameters used in the probabilistic sensitivity analysis

Supplementary appendix 6: Resource use and costs for SNP testing

Supplementary appendix 7: Breakdown of cost by category

Supplementary appendix 8: Total costs of the risk stratification strategies using resource use data from each CTA

Supplementary Appendix 1: Stages taken in costing the exemplar applications of breast cancer risk-stratification


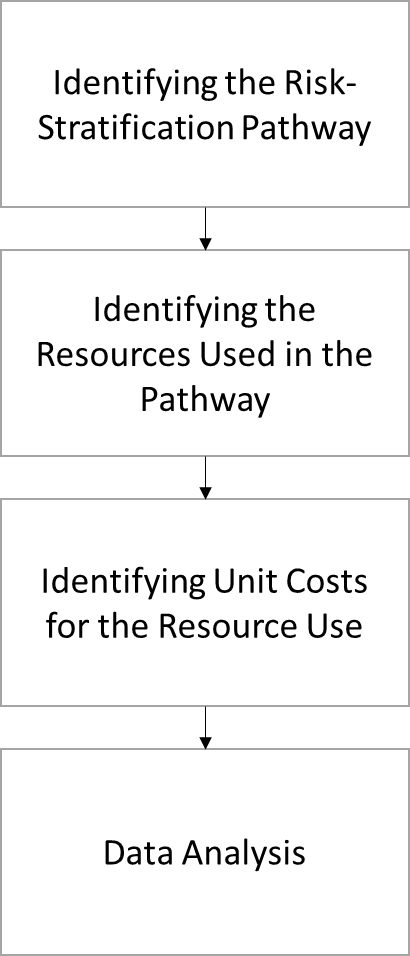


Supplementary Appendix 2: Process of single nucleotide polymorphism (SNP) testing

A saliva sample was taken at the mammography appointment and sent to a genetic laboratory for testing. DNA was isolated from saliva samples. The concentration was measured and the sample diluted accordingly.

SNP genotyping was performed using the Illumina Infinium OncoArray platform. The DNA samples were first amplified during an overnight incubation to generate a sufficient amount of sample. The samples were then fragmented, precipitated and re-suspended. Each sample was dispensed onto a BeadChip and left to hybridize overnight. Following hybridization the BeadChips were washed, and the primers extended and stained. The BeadChips were then loaded into an iScan Microarray Scanner.

After scanning the files were imported into the GenomeStudio software package where the genotypes were generated [1]. The call rate was required to be above 95% for the sample to pass. The genotypes were exported into Microsoft Excel and a polygenic risk score (PRS) calculated for each sample [2].

Supplementary appendix 3: Assumed change in risk prediction in the health service compared to the trial

It was assumed that if the strategy was implemented alongside the NHSBSP the invitation to complete an online TC questionnaire would likely be automatically generated and included with a woman’s invite to her first mammography in the NHS rather than being compiled and sent separately. It was also assumed that in the long run the implementation issues with the VBD measurement would be resolved.

Outside of the study setting, it was assumed that certain tasks could be automated to reduce required resources. In routine clinical practice, the generation of risk letters for patients would be automated upon receipt of all relevant data or after a given length of time and so administrators would not be required to do this. Women would use an online booking system to arrange risk feedback appointments rather than phone an administrator. It was also assumed that a clinician would be able to automatically generate a risk pack with information on a specific woman’s risk or to be able to view this on a computer without an administrator having to print out a pack. The study team also foresee risk feedback appointments for women predicted to be at high or moderate risk being provided by a specialist trained nurse in clinical practice as opposed to a medical oncologists or geneticists in the study. Even when considering the scenario of implementation of the risk-stratification strategy in the NHS, it was assumed that the level of uptake for risk consultations would remain the same in clinical practice.

Supplementary appendix 4: Interview schedule

Invitation to the study

How long would it take to create an invitation pack to invite women to take part in the study? Could you estimate a range on that value?

How many sheets of paper would that include?

Receiving the Tyrer-Cuzick questionnaire

What proportion of women completed an online version?

Did anyone complete the survey on the phone?

How many sheets of paper were in the paper questionnaire?

How long did it take you to enter the information from paper questionnaires onto the CRA system? And what was the range on that?

When women completed the online survey was any input required from you to process those surveys?

Volpara and SNPs

Did the entering of data from Volpara breast density measurement or SNPs require any input from you?

Creating a risk letter

How did you know when the information was ready to send to a woman?

How much time did you have to spend sending data from CRA to compiler to generate the risk letters?

How much time did it take to compile the risk letter?

How many pages were there in the risk letter?

Arranging the risk appointment

Did you have any involvement in arranging the risk appointments for women at high or moderate risk?

Other

Did you spend any time doing anything else on the project to generate women’s risk scores?

Were there any other issues which you faced in the trial which might not be faced in the NHS?

Supplementary appendix 45: Distributions of parameters used in the probabilistic sensitivity analysis

Table A4.1 shows the distributions used in the probabilistic analysis. As only a small number of experts provided estimates of proportions and resource use in this study, wide distributions were deliberately chosen to incorporate uncertainty. Where the clinical trials assistant provided estimated of resource use, each value was treated as an observation. As the correct type distribution could not be estimated based on two observations, the type was chosen based on the author’s previous experience of fitting distributions to different types of data. Where distributions were fit to two data points, the package fitdistr in the software R [3]. Where a distribution was fit to the 0.95 confidence intervals, the package rriskdstributions was used.

Table A5.1 Distributions of parameters used in the probabilistic sensitivity analysis

| **Parameter** | **Distribution** | **Hyperparameter 1** | | **Hyperparameter 2** | | **Source [reference if applicable]** |
| --- | --- | --- | --- | --- | --- | --- |
| **Proportions** |  | $\alpha$ | | $\beta$ | |  |
| Participants completing paper TC questionnaires | Beta | 10 | | 190 | | Interviews  Based on 10 paper surveys from a total of 200 |
| Paper questionnaires missing data requiring phone call | Beta | 3.5 | | 6.5 | | Interviews  Based on 3 to 4 (mind-point 3.5) out of 10 paper surveys |
| Mammography images that fail to transfer automatically | Beta | 12.61 | | 26.19 | | Interviews  Beta distribution fit to estimates given by CTAs of the proportion of image transfers that fail (25% and 40%) |
| Mammography images that require manual BIRADS data entry | Beta | 85 | | 1,915 | | Personal communication from administrator and statistician working on the PROCAS study  Based on 85 images from 2000 women |
| Women at high risk of breast cancer: TC | Beta | 734 | | 49,266 | | [4] |
| Women at high risk of breast cancer: TC+VBD | Beta | 83 | | 1,585 | | [5] |
| Women at high risk of breast cancer: TC+VBD+SNP | Beta | 123 | | 1,545 | | [5] |
| Women at moderate risk of breast cancer: TC | Beta | 4,230 | | 45,770 | | [4] |
| Women at moderate risk of breast cancer: TC+VBD | Beta | 186 | | 1,482 | | [5] |
| Women at moderate risk of breast cancer: TC+VBD+SNP | Beta | 175 | | 1,493 | | [5] |
| Woman at high risk organises risk appointment | Beta | 500 | | 173 | | [4] |
| Woman at moderate risk organises risk appointment | Beta | 68 | | 25 | | [4] |
| **Resource Use** |  | $\alpha$ | | $\theta$ | |  |
| Staff time creating invitation packs | Gamma | 2.382 | | 0.475 | | Interviews  Gamma distribution fit to estimates provided by CTAs (2 minutes and 8.03 minutes) |
| Staff time to enter data from paper surveys onto CRA | Gamma | 5.545 | | 0.261 | | Interviews  Gamma distribution fit to estimates provided by CTAs (12.5 minutes and 30 minutes) |
| Staff time to manually send breast density images | Gamma | 24.66 | | 3.95 | | Interviews  Gamma distribution fit to estimates provided by CTAs (7.5 minutes and 5 minutes) |
| Staff time to send CRA data to compiler | Gamma | 37.58 | | 4.97 | | Interviews  Gamma distribution fit to estimates provided by CTAs (5 minutes and 10 minutes) |
| Staff time to organise risk appointment | Gamma | 8.654 | | 3.846 | | Interviews  Gamma distribution fit to estimates provided by CTAs (1.5 minutes and 3 minutes) |
| Staff time to compile risk pack for clinician | Gamma | 1.547 | | 0.088 | | Interviews  Gamma distribution fit to estimates provided by CTAs (5 minutes and 30 minutes) |
| Lab staff time for preparation | Gamma | 26.7 | | 1.77 | | Personal communication  Gamma distribution fit using minimum and maximum times provided by geneticist as 95% confidence intervals |
|  |  | *a* | | *b* | |  |
| Staff time to take saliva sample | Uniform | 2 | | 5 | | Personal communication  Uniform distribution fitted to maximum and minimum of provided range (2-5 minutes) |
|  |  | $\mu$ | | $\sigma$ | |  |
| Staff time for aliquot in SNP testing | Log-normal | 3.807 | | 0.147 | | Personal communication  Log-normal distribution fit using minimum and maximum times provided by geneticist as 95% confidence intervals |
| Staff time for qubit in SNP testing | Log-normal | 4.084 | | 0.361 | | Personal communication  Log-normal distribution fit using minimum and maximum times provided by geneticist as 95% confidence intervals |
| Staff time for dilutions in SNP testing | Log-normal | 4.094 | | 0.354 | | Personal communication  Log-normal distribution fit using minimum and maximum times provided by geneticist as 95% confidence intervals |
| Staff time on day 2 of running oncoarray analysis | Log-normal | 6.109 | | 0.0329 | | Personal communication  Log-normal distribution fit using minimum and maximum times provided by geneticist as 95% confidence intervals |
| Staff time on day 3 of running oncoarray analysis | Log-normal | 6.109 | | 0.0329 | | Personal communication  Log-normal distribution fit using minimum and maximum times provided by geneticist as 95% confidence intervals |
| Number of screens conducted in each NHS trust per month | Log-normal | 6.105 | | 0.678 | | [6]  The number of screens per week conducted at each NHS trust was calculated. A log-normal distribution was fitted to this sample |
|  |  | *a* | *b* | | *c* |  |
| Staff time for incubation in SNP testing | Triangular | 60 | 60 | | 120 | Personal communication  Triangular distribution fit using minimum and maximum times provided by geneticist |
| Staff time for procedure in SNP testing | Triangular | 90 | 150 | | 150 | Personal communication  Triangular distribution fit using minimum and maximum times provided by geneticist |
| Staff time for plate layouts in SNP testing | Triangular | 60 | 60 | | 120 | Personal communication  Triangular distribution fit using minimum and maximum times provided by geneticist |
| Staff time on day 1 of running oncoarray analysis | Triangular | 120 | 120 | | 150 | Personal communication  Triangular distribution fit using minimum and maximum times provided by geneticist |
| **Other** |  | Min | | Max | |  |
| Cost of a postage stamp | Randomly assigned to one of two values | 0.66 | | 0.85 | | Randomly assigned as the cost of a 1^st^ or 2^nd^ class stamp |
| Number of mobile breast screening vans per NHS trust | Random integer between | 1 | | 3 | | Randomly assigned as a number between 1 and 3 |
| Number of times each van moves in a month | Random integer between | 2 | | 4 | | Randomly assigned as a number between 2 and 4 |
|  |  | *a* | | *b* | |  |
| Cost of Volpara breast density measurement ($US) | Uniform | 1 | | 2 | | Personal communication  Uniform distribution fitted to the minimum and maximum stated cost |

Supplementary appendix 6: Resource use and costs for SNP testing

Table A6.1: Staff time

| **Task** | **Duration (Minutes)** |
| --- | --- |
| **DNA extractions (max 20 samples)** |  |
| Prep (labels) | 15 |
| Incubation | 60 |
| procedure | 150 |
| Incubation | 0 |
| Aliquot | 45 |
| **Total minutes** | **270** |
|  |  |
| Qubit (measuring concentrations) | 60 |
| Dilutions | 60 |
| Total set-up one batch (20) | 390 |
| **Total for batches** | **1950** |
|  |  |
| **Running test** |  |
| Plate layouts (admin) | 60 |
| Day 1 Oncoarray | 150 |
| Day 2 Oncoarray | 450 |
| Day 3 Oncoarray | 450 |
| Analysis | 450 |
| **Total for Oncoarray** | **1560** |
| **Total for samples** | **3510** |
| **Total per sample** | **36.95** |

Table A6.2: Consumables

| **Item** | **Price per pack (£)** | **Pack size** | **Price per sample (£)** |
| --- | --- | --- | --- |
| DNA extractions |  |  |  |
| Oragene kits | 12 | 1 | 12 |
| PrepIT 45ml | 475 | 281 | 1.69 |
| DNA hydration solution 500ml | 233 | 1000 | 0.23 |
| 15ml tubes | 50 | 250 | 0.2 |
| 1.5ml Graduated Conical Tube, EasyGrip Screw Cap White/Blue (Sterile) | 22.8 | 250 | 0.09 |
|  |  |  |  |
| DNA quantification |  |  |  |
| Qubit assay tubes-500 | 47.41 | 500 | 0.09 |
| Qubit dsDNA BR assay kit-500 | 183.3 | 500 | 0.37 |
| 96-Well PCR Plate, Semi-Skirted, Straight Edges, natural | 37.36 | 10 | 3.74 |
|  |  |  |  |
| Oncoarrays | 1343.77 | 48 | 28.00 |
| 96-well cap mats | 111.4 | 50 | 2.23 |
| X50 deep well plates | 127.6 | 50 | 2.55 |
|  |  |  |  |
| Total |  |  | 51.73 |

Supplementary appendix 7: Breakdown of cost by category

Table A7.1: Summary of cost by category

|  | **NHSBSP as in BC-PREDICT (£)** | | | **NHSBSP as in NHS (£)** | | |
| --- | --- | --- | --- | --- | --- | --- |
| **Cost Categories** | **TC** | **TC+VBD** | **TC+VBD+SNP** | **TC** | **TC+VBD** | **TC+VBD+SNP** |
| Sending trial invitations | 4.42 | 4.42 | 4.42 | 0 | 0 | 0 |
| Receiving and entering data from the TC questionnaire | 0.88 | 0.88 | 0.88 | 0.88 | 0.88 | 0.88 |
| Volpara breast density measurement | 0 | 2.41 | 2.41 | 0 | 1.15 | 1.15 |
| SNP saliva test | 0 | 0 | 79.37 | 0 | 0 | 79.37 |
| Generating and sending the risk letter | 7.1 | 7.1 | 7.1 | 2.73 | 2.73 | 2.73 |
| Organisation and conduct of risk consultation | 4.05 | 7.01 | 8.05 | 1.07 | 1.88 | 2.18 |
|  |  |  |  |  |  |  |
| **Total** | 16.45 | 21.82 | 102.23 | 4.68 | 6.64 | 86.31 |

Supplementary appendix 8: Total costs of the risk stratification strategies using resource use data from each CTA

Table A8.1: Total costs of the risk stratification strategies in the NHS using resource use data from each CTA

|  | **Cost (£)** | | |
| --- | --- | --- | --- |
|  | **Combined** | **Expert 1** | **Expert 2** |
| **TC** | 4.68 | 4.42 | 4.93 |
| **TC+VBD** | 6.64 | 6.38 | 6.89 |
| **TC+VBD+SNPs** | 86.30 | 86.04 | 86.55 |

Table A8.2: Total costs of the risk stratification strategies in the trial using resource use data from each CTA

|  | **Cost (£)** | | |
| --- | --- | --- | --- |
|  | **Combined** | **Expert 1** | **Expert 2** |
| **TC** | 16.45 | 13.88 | 19.03 |
| **TC+VBD** | 21.82 | 19.03 | 24.63 |
| **TC+VBD+SNPs** | 102.22 | 99.33 | 105.13 |

Appendix References

[1] Illumina, GenomeStudio Software, (2022). https://emea.illumina.com/techniques/microarrays/array-data-analysis-experimental-design/genomestudio.html (accessed April 5, 2022).

[2] Microsoft, Microsoft Excel, (2010).

[3] R Core Team, R: A language and environment for statistical computing, (2019). https://www.r-project.org.

[4] D.G.R. Evans, L.S. Donnelly, E.F. Harkness, S.M. Astley, P. Stavrinos, S. Dawe, D. Watterson, L. Fox, J.C. Sergeant, S. Ingham, M.N. Harvie, M. Wilson, U. Beetles, I. Buchan, A.R. Brentnall, D.P. French, J. Cuzick, A. Howell, Breast cancer risk feedback to women in the UK NHS breast screening population, Br. J. Cancer 2016 1149. 114 (2016) 1045–1052. https://doi.org/10.1038/bjc.2016.56.

[5] A.R. Brentnall, E.M. van Veen, E.F. Harkness, S. Rafiq, H. Byers, S.M. Astley, S. Sampson, A. Howell, W.G. Newman, J. Cuzick, D.G.R. Evans, A case–control evaluation of 143 single nucleotide polymorphisms for breast cancer risk stratification with classical factors and mammographic density, Int. J. Cancer. 146 (2020) 2122. https://doi.org/10.1002/IJC.32541.

[6] NHS Digital, Breast Screening Programme, England 2019-20: Data tables, 2021. https://digital.nhs.uk/data-and-information/publications/statistical/breast-screening-programme/england---2019-20.
